# Pediatric Forearm Fracture Characteristics as Prognostic Indicators of Healing

**DOI:** 10.7759/cureus.34741

**Published:** 2023-02-07

**Authors:** Brandon W Knopp, Matthew Harris

**Affiliations:** 1 Endocrinology, Florida Atlantic University Charles E. Schmidt College of Medicine, Boca Raton, USA; 2 Orthopedic Surgery, Jupiter Medical Center, Jupiter, USA

**Keywords:** long-term follow-up after orthopedic surgery, long-term follow-up, hand-wrist radiograph, ulna fracture, radius fracture, pediatric orthopedic surgery, orthopedic surgery, pediatrics patient, pediatric forearm fracture

## Abstract

Background

This study was conducted to investigate the characteristics, complications, radiologic features, and clinical course of patients undergoing reduction of forearm fractures to better inform patient prognosis and postoperative management.

Methodology

We conducted a retrospective chart review of 75 pediatric patients treated for forearm fractures between January 2014 and September 2021 in a 327-bed regional medical center. A preoperative radiological assessment and chart review was performed. Percent fracture displacement, location, orientation, comminution, fracture line visibility, and angle of angulation were determined by anteroposterior (AP) and lateral radiographs. Percent fracture displacement was calculated as 𝐵𝑜𝑛𝑒 𝑆ℎ𝑎𝑓𝑡 𝐷𝑖𝑠𝑝𝑙𝑎𝑐𝑒𝑚𝑒𝑛𝑡 / 𝐷𝑖𝑎𝑚𝑒𝑡𝑒𝑟 × 100% = % 𝐷𝑖𝑠𝑝𝑙𝑎𝑐𝑒𝑚𝑒𝑛𝑡. The angle of angulation and percent fracture displacement were calculated by averaging AP and lateral radiograph measurements.

Results

A total of 75 cases, averaging 11.6 ± 4.1 years, were identified as having a complete fracture of the radius and/or ulna, with 66 receiving closed reduction and nine receiving fixation via an intramedullary device or percutaneous pinning. Eight (10.7%) patients experienced complications, with four resulting in a refracture and four resulting in significant loss of reduction without refracture. Fractures in the proximal two-thirds of the radius were associated with a significant increase in complications compared to fractures in the distal one-third of the radius (33.3% vs 3.6%) (p = 0.0005). Likewise, a greater fracture displacement percentage was associated with a lower risk of refracture post-reduction as those experiencing complications had over 30% greater total displacement pre-reduction compared to patients who did not experience complications such as refracture or loss of reduction (p < 0.0001). No elevated risk of complications was found based on fracture orientation, angulation, fracture line visibility, forearm bone(s) fractured, sex, age, or arm affected.

Conclusions

Our results highlight radius fracture location and percent fracture displacement as markers with prognostic value following forearm fracture. These measurements are simply calculated via pre-reduction radiographs, providing an efficient method of informing the risk of complications following forearm fracture reduction.

## Introduction

Forearm fractures are a common injury in the pediatric population, with dual fractures of the radius and ulna comprising an estimated 40% of all pediatric fractures [1,2]. These fractures typically result from indirect trauma via a fall on an outstretched hand and may involve the radius, ulna, or both [3]. Fractures involving either the radius or the ulna tend to result from direct trauma and occur less commonly than dual fractures of the forearm bones [4]. While these fractures are common, there is much uncertainty regarding the time necessary to fully heal and the risk of refracture. Current evidence suggests the overall refracture rate is between 1% and 5% with significant disparities in the risk of refracture [5-8]. As proper management of forearm fractures is crucial to regaining complete forearm function, factors that may affect healing or increase refracture risk are valuable to informing clinical care recommendations [9,10].

Fracture characteristics such as location, involvement of one or both forearm bones, and severity of injury are useful metrics for determining the risk of refracture and the timeline of recovery. Additionally, radiologic characteristics taken pre-reduction including fracture line visibility, fracture comminution, fracture orientation, bone shaft deformity, and displacement may also be useful prognostic metrics to inform care recommendations [6,7]. Although the primary utility of radiographs for forearm bone injuries is to diagnose a fracture or other injury, a greater emphasis on the radiologic characteristics of individual patient fractures may lead to more personalized medical management and prognoses. Established variables in fracture healing time include age, gender, and nutritional status. However, investigation of additional characteristics influencing forearm fracture recovery is useful to clinicians treating this injury, especially in the pediatric population.

The healing time for forearm fractures in children is variable and cannot currently be predicted reliably. Clinical estimates of fracture healing in pediatrics range from around three to eight weeks with complete stage six healing requiring over 10 months on average [11,12]. Past studies have estimated that pediatric fractures heal at a quicker rate than adult fractures, with potentially delayed initial healing and rapid recovery thereafter [12]. Given this variance in healing time and the challenges in assuring compliance in pediatric patients, more accurate assessments of healing time may improve compliance and, subsequently, clinical results. This study seeks to address this gap in knowledge by investigating the radiologic factors contributing to refracture risk and healing time in pediatric forearm fractures.

## Materials and methods

We conducted a retrospective chart review of 75 pediatric patients treated for forearm fractures between January 2014 and September 2021 in a 327-bed regional medical center. A preoperative radiological assessment and chart review was performed. Patients were evaluated for clinical progression and complications such as refracture or loss of reduction. All patients were treated by a single orthopedic surgeon and subsequently evaluated by the same surgeon at follow-up visits. Clinical progression was determined by the providing physician.

Inclusion criteria

No patients were excluded based on preexisting conditions. All included patients were age 25 or younger. Patients included had a displaced, complete fracture of the radius and/or ulna treated with closed reduction, percutaneous pinning, or intramedullary implantation with a follow-up of up to two years. Patients lost to follow-up or without pre and postoperative radiological assessment were excluded from the analysis.

Data collection

Significant blinding measures and strict inclusion criteria were followed in data collection to ensure data reliability. Preoperative radiographs were evaluated twice and measurements were compared to ensure accuracy. Radiographs and charts were reviewed separately to decrease the risk of biasing results. Data collected from patients via chart review included age at the time of surgery, gender, type of fracture reduction, time to clinical recovery, and refracture. Preoperative anteroposterior (AP) and lateral radiographs were used to determine the involvement of the radius and/or ulna, location of the fracture, angle of deformity, fracture orientation, degree of cortical displacement, comminution, and fracture line visibility. Postoperative radiographs were used to determine when radiologic fracture recovery was achieved. Patient information obtained from the database of a 327-bed regional medical center was evaluated, and all cases meeting the inclusion criteria were included. No external funding was obtained, and no clear sources of bias were identified.

Methods of analysis

**Figure 1:**
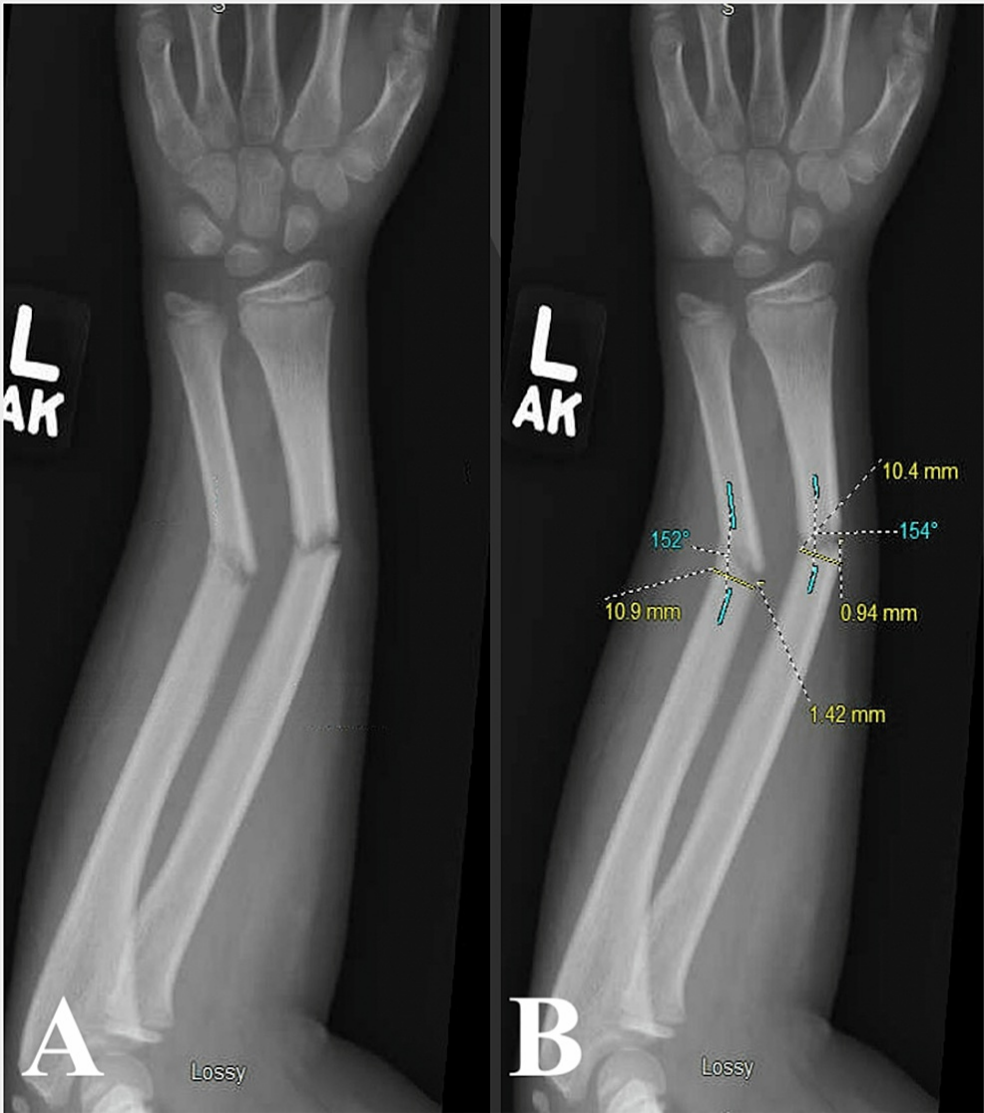
Pre-reduction radiograph in anteroposterior view with measurements unlabeled (A) and labeled (B). Yellow markings measure shaft radius and displacement, and blue markings measure the angle of deformity.

**Figure 2:**
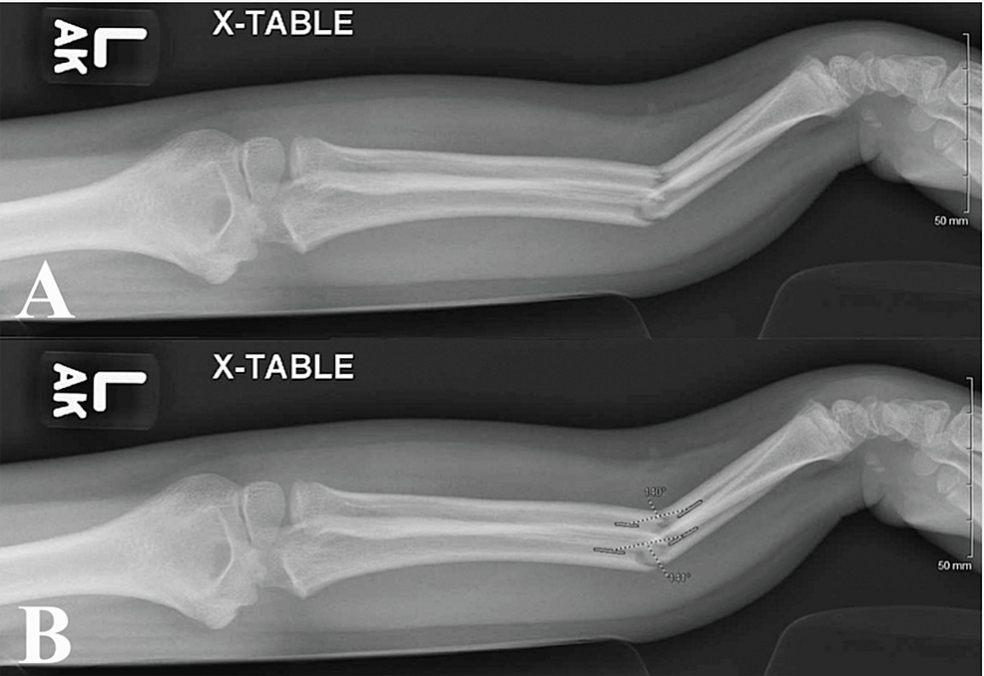
Pre-reduction radiograph in lateral view with measurements unlabeled (A) and labeled (B). Gray markings measure the angle of deformity.

The angle of deformity and cortical displacement were calculated by averaging AP and lateral radiograph measurements taken pre-reduction (Figures 1, 2). Cortical displacement was reported as a percentage of the observed cortical displacement, with a maximum of 100% reported if 100% displacement was seen in AP or lateral views. Percent cortical displacement was calculated as 𝐵𝑜𝑛𝑒 𝑆ℎ𝑎𝑓𝑡 𝐷𝑖𝑠𝑝𝑙𝑎𝑐𝑒𝑚𝑒𝑛𝑡 / 𝐷𝑖𝑎𝑚𝑒𝑡𝑒𝑟 × 100% = % 𝐷𝑖𝑠𝑝𝑙𝑎𝑐𝑒𝑚𝑒𝑛𝑡. The location of the fracture was reported as proximal one-third, middle one-third, and distal one-third of the affected radius or ulna. Fracture orientation was reported as transverse (fracture line greater than 60° relative to the long axis of the bone), short oblique (fracture line between 30° and 60° relative to the long axis of the bone), or long oblique (fracture line less than 30° relative to the long axis of the bone).

**Figure 3:**
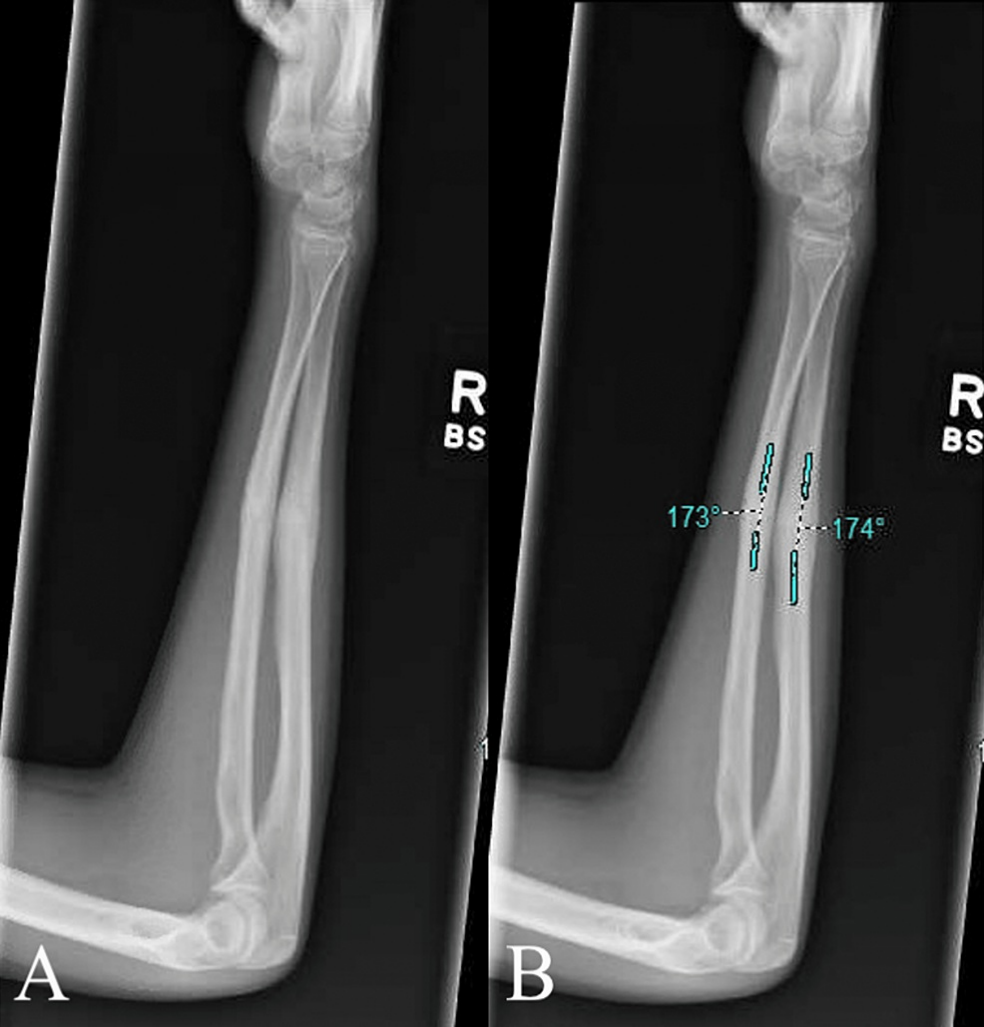
Post-reduction radiograph in lateral view with measurements unlabeled (A) and labeled (B). Blue markings measure the angle of deformity.

Recovery time was separately reported in terms of clinical recovery and radiologic recovery. Clinical recovery was determined by the physician in post-reduction follow-up visits with complete recovery allowing patients to return to full functioning. Radiologic recovery was determined by fracture union in subsequent postoperative radiographs and was defined as the presence of bridging calluses on three-fourth cortices as seen on AP and lateral radiographs (Figure 3).

Outcomes

Outcomes were reported separately regarding the presence of complications and recovery time to parse relationships between fracture characteristics and patient prognosis. Complications included refracture or significant loss of reduction.

Statistical methods

Statistical analysis was conducted by performing an independent-samples t-test and chi-squared test. The independent-samples t-test was used to analyze categorical variables, including fracture comminution, fracture line visibility, fracture orientation, recovery time, fracture location, and general patient characteristics such as gender. The chi-squared test was used to analyze continuous variables such as age, angle of deformity, and cortical displacement. The Pearson correlation coefficient test was used to assess relationships between age and variables, including pre-reduction cortical displacement and angle of deformity.

## Results

A total of 75 patients met the inclusion criteria with a complete fracture of the radius and/or ulna, preoperative radiographs, and adequate clinical follow-up. The average age was 11.6 ± 4.1 years old with 51 males and 24 females. In total, 66 patients received a closed reduction of their fracture(s), and nine received fixation via implantation of an intramedullary device or percutaneous pinning. The patients treated with operative management via hardware implantation were all over the age of 10, with no significant differences in complication rate compared to patients treated with closed reduction. A total of 31 patients were treated for radius fracture, 42 patients experienced a dual fracture of the radius and ulna, and two patients experienced an isolated ulna fracture. Among the patients with a radius fracture, with or without associated ulna fracture, three fractures were in the proximal one-third of the radius, 15 in the middle one-third of the radius, and 55 in the distal one-third of the radius. Eight patients experienced complications (10.7%), with four resulting in refracture and four resulting in significant loss of reduction without refracture. The average time to development of complications was 156 ± 264 days, with five of the seven reported times occurring within two months of the initial injury. No correlation was found between age and angle of deformity or percent cortical displacement.

Fracture in the proximal two-thirds of the radius was associated with a significant increase in complications compared to fractures in the distal one-third of the radius (33.3% vs 3.6%) (p = 0.0005). No elevated risk of complications was found based on forearm bone(s) fractured, method of reduction, sex, age, or arm affected (Table 1).

**Table 1:** Patient characteristics.

	No complications	Complications
Average age (years)	11.6 ± 4.0	12.0 ± 5.1
Male	47	4
Female	20	4
Radius	29	2
Radius and ulna	36	6
Ulna only	2	0
Left forearm	35	5
Right forearm	32	3
Closed reduction	58	7
Open reduction internal fixation	9	1
Proximal radius	1	2
Middle radius	11	4
Distal radius	53	2

The angle of deformity was an average of 23.8° ± 18.0° with no significant difference between the no complications and complications groups. Cortical displacement, reported as a percentage averaged between AP and lateral radiographs with a maximum of 100%, was 41.0% ± 40.3% on average with a significant difference between the no complications group at 45.6% ± 40.8% and complications group at 11.8% ± 14.1% (p = 0.0117). In contrast, neither fracture comminution, fracture line visibility, nor fracture orientation measured pre-reduction were significantly associated with the development of complications (Table 2).

**Table 2:** Radiologic features of radius fractures. *Excludes the two isolated ulna fractures.

	No complications	Complications	Total
Angle of deformity	23.9° ± 12.5°	23.1° ± 20.5°	23.8° ± 18.0°
Percent displacement	45.6% ± 40.8%	11.8% ± 14.1%	41.0% ± 40.3%
No fracture comminution	59	7	66
Fracture comminution	6	1	7
No fracture line visibility	18	2	20
Fracture line visibility	47	6	53
Fracture orientation (including ulna fractures)			
Transverse	58	7	65
Short oblique	4	1	5
Long oblique	5	0	5
Total	67	8	75

Recovery time was reported as the time to clinical recovery and radiologic recovery. Clinical recovery was defined based on the clinician’s assessment of bone strength recovery and ability to return to full functioning, while the radiologic recovery was defined as fracture union (presence of bridging calluses on three-fourth cortices as seen on AP and lateral radiographs) [13]. Clinical recovery and radiologic recovery, seen in Table 3, primarily occurred between one and six months, with radiologic recovery occurring slightly earlier than clinical recovery. The average time to clinical recovery was 3.5 ± 1.8 months, and the average time to radiologic recovery was 2.5 ± 1.4 months (p = 0.0003).

**Table 3:** Recovery times.

	Clinical recovery	Radiologic recovery
Less than three months	41	53
Three to six months	25	17
Greater than six months	7	1
Mean recovery time (in months)	3.5 ± 1.8	2.5 ± 1.4

Fractures in the proximal two-thirds of the radius were more likely to take more than three months to form bridging calluses on three-fourth cortices compared to fractures in the distal one-third of the radius (p = 0.0233). Other fracture characteristics were not statistically different between patients who experienced a radiologic or clinical healing time greater than or less than three months (Table 4).

**Table 4:** Radiologic recovery and fracture characteristics pre-reduction.

	Less than three months	Greater than three months
Angle of deformity	23.1° ± 16.4°	25.8° ± 23.0°
Percent displacement	38.3% ± 41.2%	56.4% ± 37.9%
Location on radius		
Proximal one-third	2	1
Middle one-third	7	7
Distal one-third	42	10
Fracture orientation (including ulna fractures)		
Transverse	47	14
Short oblique	4	1
Long oblique	2	3

## Discussion

Creating accurate timelines of fracture healing is vital to effective post-reduction management of bone fractures. The time required for complete fracture recovery is not well-described. Pediatric fractures, in particular, can have highly variable recovery rates, despite a more rapid rate of bone healing compared to adults [14]. In forearm fractures, involvement of either or both the radius and ulna can affect fracture healing due to a myriad of factors, including fracture stress and the amount of supportive alignment. This project includes a cohort of pediatric patients with complete forearm fractures in either or both forearm bones to investigate the characteristics of patients and fractures which may influence healing time. Additional prognostic factors may allow physicians to identify fracture healing time more accurately for individual patients. To achieve this goal, factors influencing recovery time must first be identified whereas future research may aim to quantify how certain fracture characteristics affect overall fracture healing time and risk of refracture.

By identifying these factors, this study seeks to create tools for increasing the accuracy of predictions regarding pediatric forearm fracture healing time and risk of refracture. In doing so, we found several variables which stood out as significantly associated with a greater risk of refracture or loss of reduction. As reported in past studies, we found that fractures in the proximal portion of the radius were at a higher risk of refracture or loss of reduction [6,7]. Potentially due to a greater degree of stress and deforming forces present in proximal radius fractures, we found a complication rate of 33.3% in proximal two-third forearm fractures compared to 3.6% in distal one-third forearm fractures (p = 0.0005). Likewise, fractures in the proximal two-thirds of the radius were more likely to take more than three months to form bridging calluses on three-fourth cortices (p = 0.0233).

We also found that patients who experienced no complications had an average of over 30% greater cortical displacement pre-reduction compared to patients who experienced refracture or loss of reduction (45.6% vs 11.8%) (p = 0.0117). This is contrary to what some may expect, as a greater cortical displacement percentage was associated with a lower risk of complications post-reduction. Conversely, cortical displacement had no significant association with recovery time. One potential hypothesis for this is that larger degrees of cortical displacement lead to more robust healing responses and are associated with conditions more conducive to bone repair, such as a larger fracture surface area exposed for callus formation post-reduction. While these conditions may lead to increased fracture strength recovery and lower refracture risk, they would not impact healing speed. In any case, future research is necessary to expound on our current understanding.

As the radiologic factors investigated are determined pre-reduction, this approach allows simple measurements to inform patient expectations more thoroughly even before reduction is complete. The reason why these factors have potential prognostic value may relate to the process of fracture healing with impacts on the clearance of bone fragments and subsequent callus formation and bone remodeling. Conversely, they may contribute to healing time and refracture risk via an unknown mechanism. Regardless, prognostic factors in pediatric forearm fracture recovery are valuable and may be a vital component of future attempts to develop more accurate timelines of pediatric fracture healing, as advocated by Messer et al. (2020) [14].

This study has several limitations including a small sample size, reliance on retrospective data, and inability to account for patient risk factors such as hypocalcemia, low vitamin D, and poor compliance with activity recommendations. However, the study provides important information on radiologic factors which can inform a clinical prognosis and care recommendations. The results concur with previous research which indicates fracture location on the radius impacts clinical progression. Additionally, this study identifies cortical displacement as a notable metric in determining a likely clinical progression as well.

## Conclusions

This study is an investigation of preoperative forearm fracture characteristics which may inform injury prognosis, clinical progression, and risk of complications including refracture or loss of reduction. In evaluating potential prognostic factors, both qualitative and quantitative measurements were taken into consideration by utilizing clinical evaluations and radiologic assessments. While qualitative evaluations are vital in determining patient prognosis, this study focused primarily on quantitative measures for the reproducibility of findings. Likewise, we sought to create a wide scope of analysis by including both clinical determinations and radiologic assessments of fracture healing. In doing so, we identified increased percent cortical displacement and forearm fracture location to be noteworthy pre-reduction markers that may be able to inform patient progression following forearm fracture.
